# Tracking of Zinc Ferrite Nanoparticle Effects on Pea (*Pisum sativum* L.) Plant Growth, Pigments, Mineral Content and Arbuscular Mycorrhizal Colonization

**DOI:** 10.3390/plants10030583

**Published:** 2021-03-19

**Authors:** Reda E. Abdelhameed, Nagwa I. Abu-Elsaad, Arafat Abdel Hamed Abdel Latef, Rabab A. Metwally

**Affiliations:** 1 Botany and Microbiology Department, Faculty of Science, Zagazig University, Zagazig 44519, Egypt; 2 Physics Department, Faculty of Science, Zagazig University, Zagazig 44519, Egypt; nagwa.ibrahim@yahoo.com; 3 Department of Biology, Turabah University College, Turabah Branch, Taif University, P.O. Box 11099, Taif 21944, Saudi Arabia

**Keywords:** arbuscular mycorrhizal fungi, *Pisum sativus*, plant growth, translocation factor, zinc ferrite nanoparticles

## Abstract

Important gaps in knowledge remain regarding the potential of nanoparticles (NPs) for plants, particularly the existence of helpful microorganisms, for instance, arbuscular mycorrhizal (AM) fungi present in the soil. Hence, more profound studies are required to distinguish the impact of NPs on plant growth inoculated with AM fungi and their role in NP uptake to develop smart nanotechnology implementations in crop improvement. Zinc ferrite (ZnFe_2_O_4_) NPs are prepared via the citrate technique and defined by X-ray diffraction (XRD) as well as transmission electron microscopy for several physical properties. The analysis of the XRD pattern confirmed the creation of a nanocrystalline structure with a crystallite size equal to 25.4 nm. The effects of ZnFe_2_O_4_ NP on AM fungi, growth and pigment content as well as nutrient uptake of pea (*Pisum sativum*) plants were assessed. ZnFe_2_O_4_ NP application caused a slight decrease in root colonization. However, its application showed an augmentation of 74.36% and 91.89% in AM pea plant shoots and roots’ fresh weights, respectively, compared to the control. Moreover, the synthesized ZnFe_2_O_4_ NP uptake by plant roots and their contents were enhanced by AM fungi. These findings suggest the safe use of ZnFe_2_O_4_ NPs in nano-agricultural applications for plant development with AM fungi.

## 1. Introduction

Agriculture is the economic backbone of many countries, and in developing countries it is considered the main livelihood of the rural population [1] as it is the chief food source and expected to feed the ever-rising population worldwide [2]. Nevertheless, poor soil fertility is a chief restriction on crop productivity. Nowadays, nanotechnology is expected to be the base of several biotechnological innovations in the 21st century and is regarded as the upcoming industrial revolution [3] as its application can be observed in innumerable fields (pharmacy, medicine, materials science, environmental protection and agriculture, etc.). Nanotechnology applications in the food and agriculture sector attract attention where nanoagrochemicals, for instance, nanofertilizers, nanopesticides, nanoparticles (NPs)-based growth stimulators and nanocarriers, are potentially more effective and pose a lower risk of environmental contamination than their conventional analogues [4,5]. NPs are known as a stimulating agent for plant growth modulating the physiological, biochemical and physicochemical pathways, such as photosynthesis and nutrient uptake. Additionally, NPs accumulation in plants is of great significance not only for their prospective effects on plant development and growth but also for the health of the human body [6]. Whereas, nanofertilizers or nanoencapsulated nutrients have the properties to effectively release nutrients on demand, which regulate plant growth and increase target activity [7,8,9]. Conversely, some reports documented neutral or negative responses to plants exposed to NPs [10].

Microorganisms in soil play key roles in nutrient cycling and contribute to increasing plant growth and developing the plant’s health; they are also responsible for a vast number of soil functions [11,12]. As a representative, arbuscular mycorrhizal (AM) fungi, possibly the most important symbioses on earth, can form a mutualistic symbiosis with the roots of over 90% of land plants. They play a key role in plant growth promotion directly by providing nutrition and/or indirectly by protecting against biotic and abiotic stresses [13,14,15,16]. AM fungi as an example of soil microorganisms that can be affected by, and exposed to, NPs that are either intentionally liberated into the environment (NP containing amendments besides nanoagrochemicals) or reach into the soil as nanomaterial pollutants [17]. AM fungi can mediate the effects of the heavy metals on their hosts, allowing some plants to grow in soils with excess toxic metals such as Zn [18]. Additionally, AM fungi help alleviate metal stress in *Phragmites australis* and *Iris pseudacorus* by transforming cationic copper into metallic NPs, which implies that AM fungi may impact metal NPs effects on plants [19].

Of particular note, information about the prospective influence of NPs on the functioning of AM symbiotic associations is quite limited. Primary studies showed that metal NPs application may exert both positive and adverse effects [20] on AM fungi due to their accumulation. Additionally, metal oxides NPs such as iron oxide (FeO) NPs and silver (Ag) NPs differently influenced AM fungi, and the consequential effects of AM fungi on plant growth were primarily described by [21]. Similarly, AM root colonization was decreased by Fe_3_O_4_ NPs or Ag NPs [21,22]. The pattern that drives this variability in the responses of plant associations with AM fungi to NPs varies according to the NPs’ properties, concentration, AM fungal species and characteristics of the soil in which the AM fungi are living and interacting with plant roots [17].

Ferrite NPs are interesting materials due to their rich physical, structural, electrical and magnetic properties [23]. They have an enormous impact on the applications of magnetic materials as they are wear- and oxidation-resistant [24,25]. Iron-based magnetic spinel ferrite NPs with the general formula AFe_2_O_4_ (A = Ni, Co, Zn, Cu, Mg, Mn, etc.) are intensely used in many sectors and purposes, for instance, for medical implementations and the remediation of soil and water [26]. These magnetic NPs are selected for some specific applications because they can be easily traced in the organism and can be directed externally by magnets [27,28,29]. Among the magnetic NPs, zinc ferrite (ZnFe_2_O_4_) has involved much consideration due to its moderate magnetic saturation, low coercivity, chemical and thermal stability, mechanical hardness and high electromagnetic performance [30,31]. Similarly, [23,32] studied the effect of CuFe_2_O_4_ NPs and CoFe_2_O_4_ NPs on cucumber and tomato plants, respectively, and stated their nutritive special effects on plants. The particle size of the nanofertilizer is less than the pore size of the leaves and roots, thereby enhancing the strength of the nanofertilizers’ penetration into the plant when applied evenly on the plant surface and thus increasing the quality of the nutrient usage while reducing the cost of the input [9,33]. Otherwise, [12] documented that iron oxide magnetic NPs exert inhibitory effects on the biomass of maize.

The green pea (*Pisum sativus* L.) is one of the broadly used legumes in healthy diets [34] because of its high protein content, essential amino acids such as lysine and leucine, minerals such as K, P, Ca, Cu, Fe and Zn and vitamins. To our knowledge, no study has yet been conducted to explore the consequence of ZnFe_2_O_4_ NPs on AM fungal colonization and their dual role (AM fungi and ZnFe_2_O_4_ NPs) in green pea growth performance as well as chlorophyll content analyses. Furthermore, Zn and Fe concentrations and translocation in the plant were studied in both AM and non-AM treated plants. As the impact of NPs is based on their concentration, size and distribution, we firstly investigated the structural and magnetic properties of ZnFe_2_O_4_ NPs.

## 2. Results

### 2.1. X-ray Diffraction (XRD) Analysis

**Figure 1:**
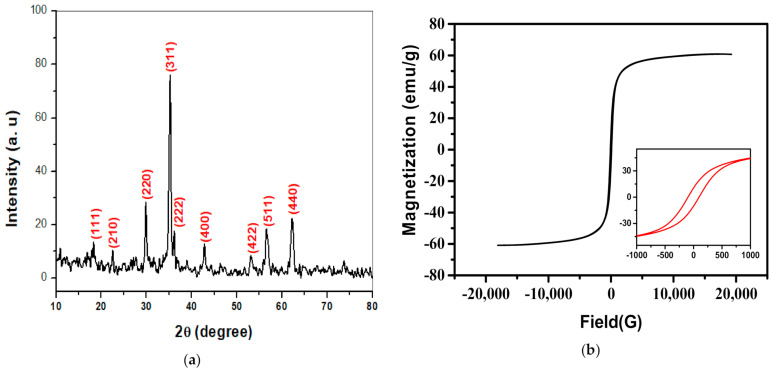
Characterization of ZnFe_2_O_4_ NPs powder; (**a**) X-ray diffraction (XRD) and (**b**) magnetic hysteresis (M-H) loop.

X-ray diffraction (XRD) analysis was used to recognize the crystal structure of NPs as presented in Figure 1a. The lattice constant was computed utilizing Equation (1) [35]. The crystallite size (t) has been computed employing the Debye–Scherrer expression [36] as Equation (2):

(1) $$a=d_{\left(hkl\right)}\sqrt{\left(h^{2}+k^{2}+l^{2}\right)}$$

(2) $$t=\frac{0.9\;\lambda }{\beta cos\theta }$$

where *θ* and *λ* are the diffraction angle as well as the wavelength for the target used, respectively. Moreover, *β* is the full width at half-maximum of the diffraction peaks. The crystallite size was estimated for the three high intense diffraction peaks (220), (311) and (440). It was discovered that the crystallite size (*t*) was 25.4 nm, which proves the nanocrystalline nature of zinc ferrite.

### 2.2. Magnetic Hysteresis (M-H) Measurements

The magnetic hysteresis loop of ZnFe_2_O_4_ NPs powder at room temperature was shown in Figure 1b. The attained coercivity (H_c_) and saturation magnetization (M_s_) were 96.56 G and 60.21 emu/g, respectively.

### 2.3. Transmission Electron Microscope (TEM)

**Figure 2:**
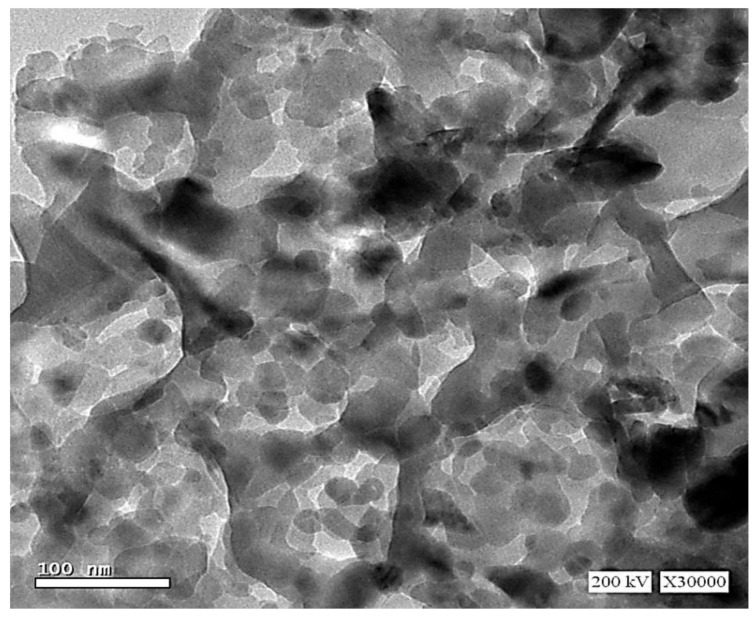
Transmission electron microscope (TEM) image of ZnFe_2_O_4_ NPs powder.

The TEM micrograph of ZnFe_2_O_4_ NPs powder was displayed in Figure 2. The particle size was around 20–30 nm. It can be noticed that NPs were well scattered beside some agglomeration in a few crystallites.

### 2.4. Effects of ZnFe_2_O_4_ NPs on Plant Growth Traits

**Figure 3:**
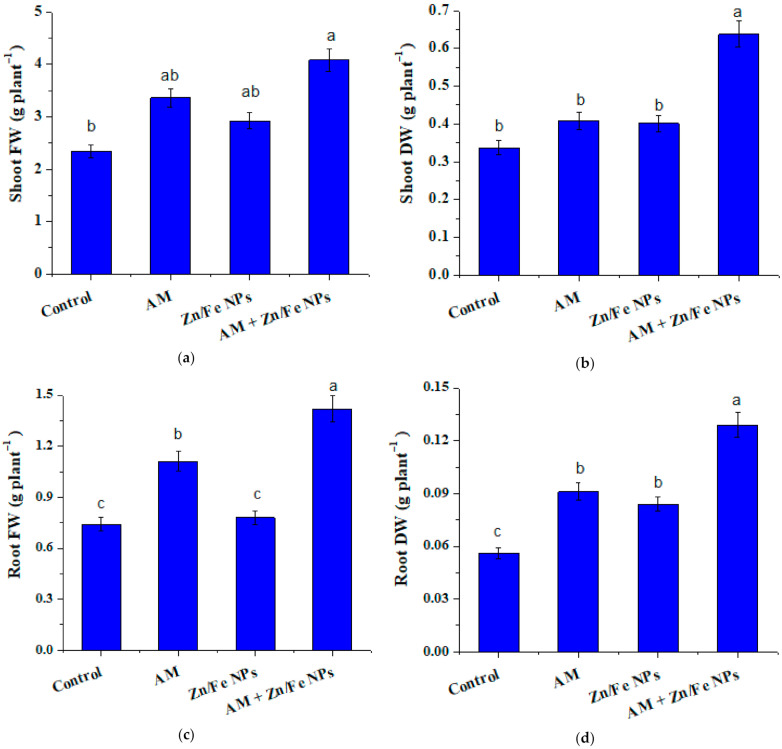
ZnFe_2_O_4_ NPs and arbuscular mycorrhizal (AM) fungal effects on shoot fresh weight (**a**), shoot dry weight (**b**), root fresh weight (**c**) and root dry weight (**d**) of pea plants. Control: represents non-treated pea plants; AM: represents pea plants inoculated with AM fungi; Zn/Fe NPs: pea plants treated with ZnFe_2_O_4_ NPs, and AM + Zn/Fe NPs: represents pea plants dually treated with AM fungi and ZnFe_2_O_4_ NPs. Data are the mean of three replicates ± standard error (n = 3). Different letters above bars indicate a significant difference between treatments using ANOVA followed by Duncan’s multiple range test (*p* < 0.05). FW, fresh weight; DW, dry weight.

**Figure 4:**
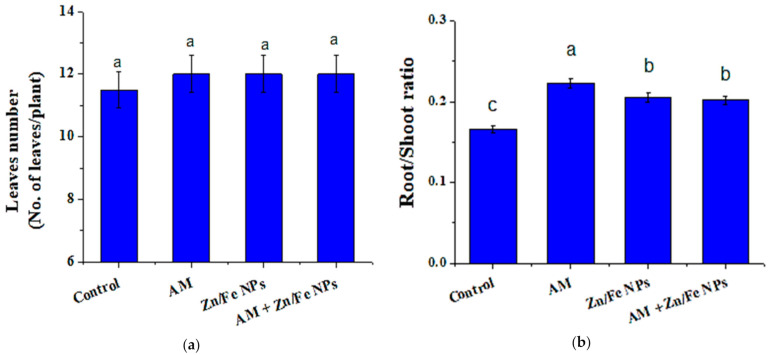
ZnFe_2_O_4_ NPs and AM fungal effects on: (**a**) leaves number and (**b**) root/shoot ratio of pea plants. Control: represents non-treated pea plants; AM: represents pea plants inoculated with AM fungi; Zn/Fe NPs: pea plants treated with ZnFe_2_O_4_ NPs and AM + Zn/Fe NPs: represents pea plants dually treated with AM fungi and ZnFe_2_O_4_ NPs. Data are the mean of three replicates ± standard error (n = 3). Different letters above bars indicate a significant difference between treatments using ANOVA followed by Duncan’s multiple range test (*p* < 0.05).

Uptake and translocation of ZnFe_2_O_4_ NPs in the pea plant body may alter a number of morphological and physiological parameters. The effects of AM fungal inoculation and ZnFe_2_O_4_ NPs applications on pea growth traits, for instance, fresh and dry weight, are depicted in Figure 3. As compared to control (non-inoculated with AM fungi or ZnFe_2_O_4_ NP-treated) plants, AM or ZnFe_2_O_4_ NPs single application revealed a substantial increase (*p* ≤ 0.05) in all pea growth criteria except leaves number, where the increase was not significant (Figure 4a). It is worth mentioning that the magnitude of such an increase was more pronounced (Table 1) with their combination (ZnFe_2_O_4_ NPs + AM inoculation). It is interesting to point out that pea plants dually applied with AM and ZnFe_2_O_4_ NPs showed an enhancement of 74.36% and 91.89% in shoot and root fresh weight and 89.51% and 130.18% in shoot and root dry weight, respectively, versus the control. Another worth mentioning was that the root/shoot ratio of AM or ZnFe_2_O_4_ NPs pea plants singly or dually treated was higher than control (Figure 4b).

### 2.5. AM Fungal Colonization Rate

**Figure 5:**
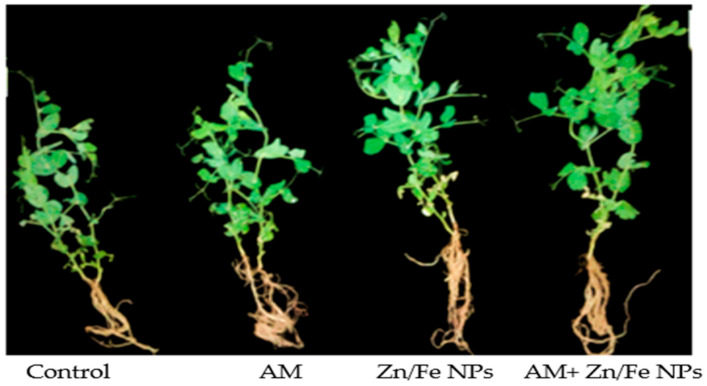
Photographs of pea plants showing the differences between root and shoot of control (non-treated pea plants); AM (pea plants inoculated with AM fungi); Zn/Fe NPs (pea plants treated with ZnFe_2_O_4_ NPs); AM+ Zn/Fe NPs (pea plants dually treated with AM fungi and ZnFe_2_O_4_ NPs) pea plants.

**Figure 6:**
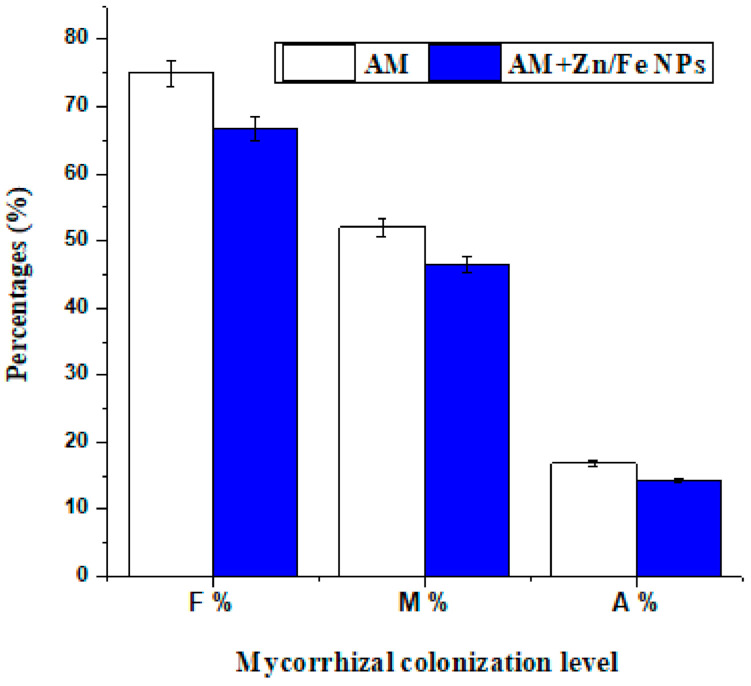
ZnFe_2_O_4_ NP effect on mycorrhizal colonization level of pea plant roots. F%: represents frequency of mycorrhizal colonization; M%: represents intensity of mycorrhizal colonization; A%: represents arbuscular frequency of pea plant roots. AM: represents pea plants inoculated with AM fungi and AM + Zn/Fe NPs: represents pea plants dually treated with AM fungi and ZnFe_2_O_4_ NPs.

Mycorrhizal colonization levels (frequency (F%), intensity (M%) of mycorrhizal colonization besides arbuscular development (A%)) in pea root tissues to some extent were affected with ZnFe_2_O_4_ NP application; its application slightly reduced the rate of root colonization but the results were not statistically significant. Despite that, AM fungi in ZnFe_2_O_4_ NP-treated plants still function with their strength as is evident in the enhancement that occurred in pea growth traits (Figure 3, Figure 4 and Figure 5). Furthermore, the root colonization of pea plants singly treated with AM was above 70% (Figure 6).

### 2.6. Photosynthetic Pigments Content

The effect of ZnFe_2_O_4_ NPs and AM fungal application on pigment content as a measure of photosynthetic efficiency of pea plants was investigated in Table 2. Results revealed an increase in total chlorophyll content, and its fractions in pea leaves with AM fungi and ZnFe_2_O_4_ NPs applications and the magnitude of such proliferation reached its highest level with their dual application as shown in two-way ANOVA analysis (Table 1). Whereas, chlorophyll a content in pea leaves dually treated with AM fungi and ZnFe_2_O_4_ NPs was 1.729 ± 0.091 mg/g FW, followed by those singly treated with ZnFe_2_O_4_ NPs (1.549 ± 0.082 mg/g FW) or AM-inoculated (1.497 ± 0.079 mg/g FW), compared to the control (1.441 ± 0.076 mg/g FW). It was revealed that pea plants dually applied with AM and ZnFe_2_O_4_ NPs showed an improvement of 65.37%, 36.34% and 30.54% in chlorophyll b, total chlorophylls and total pigments, respectively, versus the control (Table 2).

### 2.7. Fe and Zn Content and Their Translocation in Plant Tissues

**Figure 7:**
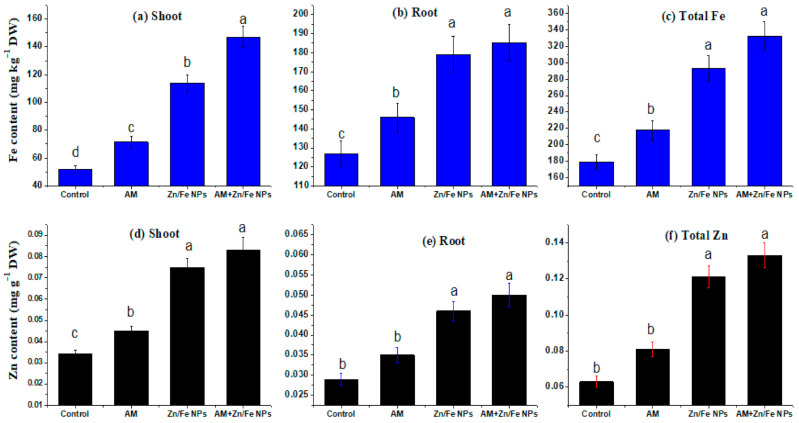
ZnFe_2_O_4_ NPs and AM fungal effects on Fe content (mg/kg DW) (**a**–**c**) and Zn content (mg/g DW) (**d**–**f**) in shoot, root and their total contents in pea plants respectively. Control (non-treated pea plants); AM (pea plants inoculated with AM fungi); Zn/Fe NPs (pea plants treated with ZnFe_2_O_4_ NPs) and AM+ Zn/Fe NPs (pea plants dually treated with AM fungi and ZnFe_2_O_4_ NPs) pea plants. Data are the mean of three replicates ± standard error (n = 3). Different letters above bars indicate a significant difference between treatments using ANOVA followed by Duncan’s multiple range test (*p* < 0.05). DW, dry weight.

To better recognize the concentration of ZnFe_2_O_4_ NPs in pea shoot and root and their migration from root to shoot, inductively coupled plasma mass spectrometry (ICP–MS) analysis was conducted. Overall, ICP–MS analysis revealed an increase in Zn and Fe content in shoot and root of peas subjected to ZnFe_2_O_4_ NPs, confirming the translocation of these nanomaterials from root to shoot. Moreover, the results showed that the Zn and Fe concentrations and migration in the pea shoot and root were influenced by ZnFe_2_O_4_ NPs, AM fungal application and the interactions between them (Figure 7 and Table 1). As expected, ZnFe_2_O_4_ NPs had the most profound effects on plant Zn and Fe concentrations as compared to AM inoculation. Whereas, ZnFe_2_O_4_ NPs addition increased both pea shoot and root Zn and Fe concentrations.

**Figure 8:**
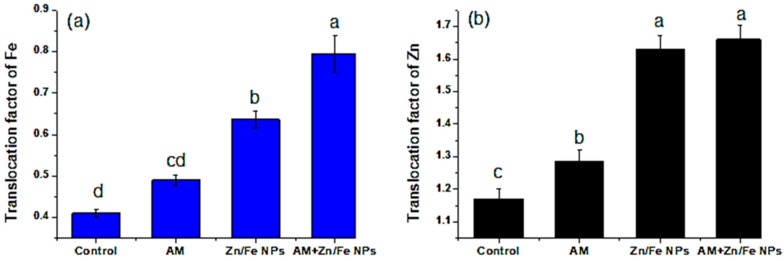
ZnFe_2_O_4_ NPs and AM fungal effects on the translocation factor of Fe (**a**) and Zn (**b**) of the control (non-treated pea plants); AM (pea plants inoculated with AM fungi); Zn/Fe NPs (pea plants treated with ZnFe_2_O_4_ NPs) and AM+ Zn/Fe NPs (pea plants dually treated with AM fungi and ZnFe_2_O_4_ NPs) pea plants. Data are the mean of three replicates ± standard error (n = 3). Different letters above bars indicate a significant difference between treatments using ANOVA followed by Duncan’s multiple range test (*p* < 0.05).

AM inoculation alone did not exert significant effects on root Zn and Fe but showed marked interactive effects with ZnFe_2_O_4_ NPs on the shoot and root Zn and Fe concentrations (Figure 7). Another interesting aspect of our results was that pea plants dually treated with ZnFe_2_O_4_ NPs and AM fungi harbor the highest Fe (180.53 ± 0.98 and 140.72 ± 0.779 mg/kg DW) and Zn content (0.050 ± 0.003 and 0.083 ± 0.006 mg/g DW) in their root and shoot, respectively. AM fungi can increase the bioavailability of ZnFe_2_O_4_ NPs and sequester the released Zn in soil, and then increase Zn uptake by plants and their transport from soil to roots and from roots to shoot. Additionally, the shoot/root Zn and Fe ratio, which indicates their internal translocation, increased with AM inoculation and ZnFe_2_O_4_ NPs (Figure 8).

## 3. Discussion

The ZnFe_2_O_4_ NPs are formed in a single-phase cubic spinel structure with the main reflection planes (111), (210), (220), (311), (222), (400), (511) and (440). It is found that the lattice parameter equals 0.845 nm ± 0.001, which was consistent with that reported previously [37]. The coercivity value was small, denoting the excellent soft magnetic properties for the present sample. The agglomeration in the TEM micrograph can be due to magnetic dipole interaction between ferric ions [38]. Moreover, the obtained particle size was in good agreement with that computed by the Scherrer formula from XRD results.

Plants such as peas suffer from nutrient deficiency stress when the availability of soil nutrients and/or the quantity of nutrients absorbed is below that required to support metabolic processes. This can be due to an inherently low soil nutrient status or low soil nutrient mobility [39]. Consequently, a significant proportion of people are micronutrient deficient (especially Fe and Zn). Zn concentration in soil solution depends greatly on soil pH and declines to very low levels at high soil pH [40]. Alkaline soils such as the soil used in our study may be zinc-rich, and plants may not take it up under these conditions as Zn becomes closely linked to the CaCO_3_ present in the soil [41]. It is reported [42] that rice, wheat and soybean plants exposed to higher CO_2_ levels would accumulate less Zn. As a result, it is desirable to use new techniques to boost plant growth that harbour higher levels of micronutrients such as Fe and Zn [43]. One of these new technologies is the application of NPs where [44] used ZnO NPs to determine their effect on maize compared to ZnSO_4_ application and reported that ZnO NPs improved yield and Zn content compared to ZnSO_4_. Additionally, AM fungi can increase the accumulation of many nutrients, including Zn [15].

Balanced nutrient management and soil enrichment are very important for improving crop productivity [45]. AM fungi are an indispensable constituent of the soil ecosystem that are active in the transformation and/or degradation of a wide variety of pollutants to sustain soil productivity and ecological functions [46]. Nevertheless, the roles of AM fungi in NPs–plant interactions have not been well examined. Therefore, we speculate that their participation could change our knowledge regarding the biological effect of NPs on plant systems.

Our results of AM fungal inoculation and ZnFe_2_O_4_ NP applications on pea growth traits are consistent with [47] who reported an increase in spinach growth when treated with TiO_2_ NPs. Additionally, ref. [48] conveyed that a low ZnFe_2_O_4_ NP concentration encourages *Chlorella pyrenoidosa* growth rate. Interestingly, ref. [49] showed that ZnO NPs significantly improved the seedling growth of the wheat plant. These proliferations might be due to the adsorption of these NPs on the cell surface and their beneficial effects on plants [50,51].

Moreover, the positive impact of ZnFe_2_O_4_ NPs on growth parameters (Figure 3a,c) can be attributed to the fact that ZnFe_2_O_4_ NPs may supply essential micronutrient Zn for plant growth [51,52], but turn out to be poisonous if they release excess Zn beyond the plant’s necessities [53]. Opposing our results, ref. [54] stated a reduction of ∼20 and 80% in Arabidopsis growth with 200 and 300 mg/L ZnO NPs. Additionally, higher doses of ZnO NPs inhibited plant growth of sweet sorghum, while a low ZnO NPs dose was non-phytotoxic [53]. According to our results, the substantial proliferation in most of the growth parameters as a result of AM fungal inoculation might be due to the superb abilities of these fungi in improving both physiological as well as morphological mechanisms and the uptake of immobile nutrients such as P, Zn and Cu through extraradical mycorrhizal mycelia that aid the acquisition of nutrients at distances plant roots cannot reach [55,56,57,58]. These findings were earlier reported by [59,60,61] in pepper, cowpea and trigonella, respectively.

Contrary to our results, ref. [10] found that ZnO NPs applied at a concentration of 800 mg/kg decreased shoot and root dry weights of AM maize plants. Our results of the root/shoot ratios (R/S) of AM or ZnFe_2_O_4_ NP-treated pea plants were in line with [14,62] who stated that AM fungi caused an increase in R/S ratios as compared to the control. Collectively, our findings point out the beneficial effects of AM and ZnFe_2_O_4_ NP applications on each other.

It was found that AM colonization and extraradical hyphal growth were suppressed when plants were grown with micronutrients (Fe, Zn, Cu and Mn) especially at a high level [63,64]. Accordingly, ref. [65] found that excess Zn additions strongly reduced AM colonization in tomato plant roots. Moreover, ref. [63] revealed that the high concentrations of trace metals can severely repress AM spore germination and root colonization in clover plant roots. It is reported [64] that both internal AM root colonization and extraradical hyphae developed in soil were more sensitive to high levels of micronutrients in the maize plant.

Even though few investigations examined the influence of metal-NPs on AM fungal colonization, the results are contradictory [10,21]. Using a sand culture microcosm experiment, ref. [21] found that the AM colonization rate of clover roots augmented with the addition of 0.032–3.2 mg/kg FeO NPs or 0.01–1 mg/kg Ag NPs. Conversely, ref. [10] reported a decrease in maize root colonization rates at higher ZnO NP concentrations (800, 1600 and 3200 mg/kg). Our present experiment shows different results. Consistent with our results, [10] found that at 400 mg/kg, the ZnO NP colonization rate of AM maize plant roots did not change significantly, although it markedly lessened at higher doses. Hence, there was a negative relationship between ZnFe_2_O_4_ NPs and root colonization. These findings denote that NPs could influence the distribution and AM fungal community composition. To our knowledge, this is the first report of AM fungal response to ZnFe_2_O_4_ NPs in soil habitats.

ZnFe_2_O_4_ NPs and AM fungal application caused amplification in pigment content of pea plants which may be ascribed to increased stomatal conductance, transpiration rate and carbon assimilation or the increase in P and Mg^2+^ uptakes using extraradical hyphae of mycorrhiza which are essential constituents necessary for the photosynthesis [66]. The increase in chlorophyll content as a result of ZnFe_2_O_4_ NPs application is consistent with the findings of [47,67] in spinach and wheat with TiO_2_ NPs applications.

A similar augmentation of maize pigments with iron oxide NPs has been reported by [51,68]. Aditionally, ref. [28] reported a gradual increase in chlorophyll a, b and carotenoids content in barley with NiFe_2_O_4_ NPs. Contradictory to our results, ref. [54] reported a reduction of more than 50% in Arabidopsis chlorophyll contents treated with 300 mg/L ZnONPs. The stimulatory effect of ZnFe_2_O_4_ NPs on photosynthesis might be due to Zn and Fe being essential micronutrients for plant metabolism and their involvement in chlorophyll biosynthesis [29]. The maximum increase in the pigment content of pea leaves dually treated with AM fungi and ZnFe_2_O_4_ NPs was in harmony with the results of [10] in maize plants treated with 400 mg/kg of ZnONPs and inoculated with AM fungi. Whereas, AM pea plants sequester more Zn in their mycorrhizal structure and improve Mg uptake, leading to augmentation in chlorophyll concentrations, consequently increasing photosynthate production as well as plant progress [69].

Fe and Zn are indispensable micronutrients for plant growth contributory to physiological processes such as photosynthesis, the production of phytohormones and chlorophyll formation, and their deficiency cause some substantial nutrient imbalances and ultimately lessens the amount as well as the quality of the crop product [39]. Although the scientific investigation of NP uptake and accumulation in plants is still in the early stages, recent publications have categorized advances in the area of NP toxicology along with uptake via plants.

Our findings of increased Zn and Fe concentrations in pea shoots and roots are comparable to the findings of [28] who stated that Fe and Ni elements steadily increased by increasing NiFe_2_O_4_ NP concentrations in *Hordeum vulgare*. A similar pattern was observed for Cu, Co and Fe uptake in cucumber and tomato plants treated with CuFe_2_O_4_ NPs and CoFe_2_O_4_ NPs [23,32]. Moreover, ref. [70] showed the release of Fe ions in an aqueous solution from the surface of magnetic Fe_2_O_3_ NPs. ZnFe_2_O_4_ NPs might be similar to ZnO NPs in that they continuously release Zn to the soil solution to replenish those scavenged by roots, as [71] stated. Additionally, some NPs in the plant tissues might be degraded or changed at the end [28,72,73]. In concert with those findings, the present study pointed out the possible degradation or liberation of Zn and Fe elements in the plant tissues. Nevertheless, the mechanism of a probable degradation could not be recognized up till now.

Our result of enhanced Zn uptake by AM fungi is divergent from [10,53] in the shoot and root of maize and sweet sorghum plants at all ZnO NP doses with AM inoculation. Additionally, the increase in the shoot/root Zn and Fe ratio with AM inoculation and ZnFe_2_O_4_ NPs is consistent with [74] who stated that NPs were taken up by the plant roots and translocated to the aerial organs including the leaves of a pumpkin (*Cucurbita maxima*); this could be due to their smaller particle size. Additionally, our results showed further augmentation due to the joined interaction between ZnFe_2_O_4_ NPs with AM application. This result conflicted with the findings of [10] in maize plants.

## 4. Materials and Methods

### 4.1. Synthesis of ZnFe_2_O_4_ NPs

**Figure 9:**
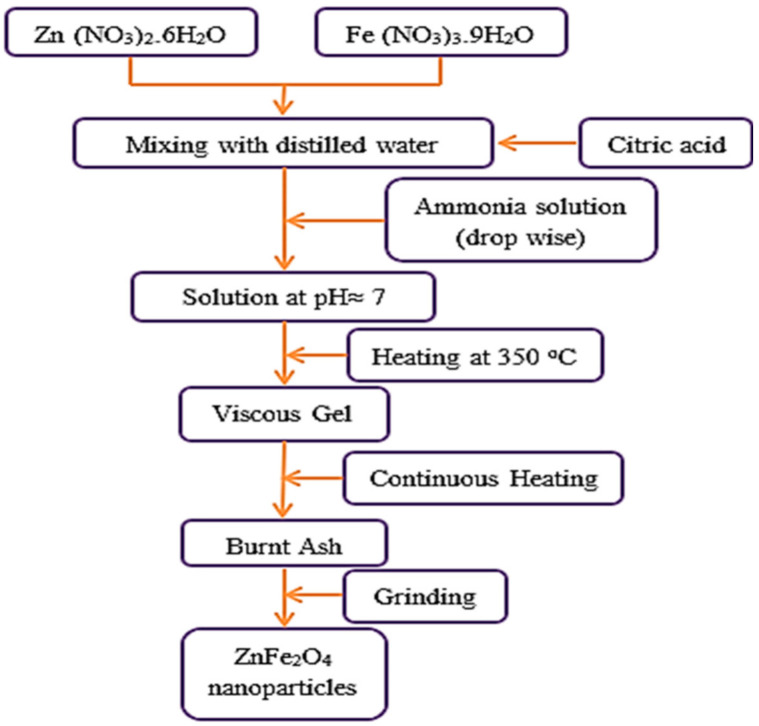
A flow chart for ZnFe_2_O_4_ NP preparation.

ZnFe_2_O_4_ NPs were synthesized via the citrate method. Analytical grade Zn (NO_3_)_2_.6H_2_O, Fe (NO_3_)_3_.9H_2_O and citric acid were used as starting reagents. The reagents were solved in de-ionized water at a ratio of 1:1 of nitrates to citric acid. Then, a solution of ammonium was gradually dropped into the solution to change the pH to ≈7.0. The mixed solution was stirred constantly at 120 °C until a viscous liquid was reached. The sol was heated to 350 °C, then ignited and burnt spontaneously. Finally, the resultant ashes were thoroughly milled in a mortar to produce excellent NPs. Figure 9 shows a flow chart for the synthesis of ZnFe_2_O_4_ NPs.

### 4.2. Nanoparticle Characterization

The X-ray diffraction (XRD) was examined at room temperature on an X-ray diffractometer, using CuK_α_ radiation (type PHILIPS X’pert Diffractometer) and the ZnFe_2_O_4_ NPs were observed through a transmission electron microscope (TEM) model, Jeol (*JEM-1230,* Tokyo, Japan). The magnetization of ZnFe_2_O_4_ NPs was measured using a Vibrating Sample Magnetometer with a maximum magnetic field of 20 kOe at room temperature.

### 4.3. Preparation of ZnFe_2_O_4_ NPs Suspension

Suspension of ZnFe_2_O_4_ NPs was prepared at a concentration of 5 μM in distilled, deionized water. The suspensions were sonicated for 4 h in a bath sonicator (Branson’s Model B200 ultrasonic) to ensure distribution of the NPs and to avoid aggregation and agglomeration.

### 4.4. Soil, Seeds, Pot Culture and Growth Condition

The soil used for plant growth was collected from the top layer of the field (0–15 cm depth) at Sharkia Governate. The soil was disinfected (2% formaldehyde) to destroy indigenous AM fungi, after passing through a 2 mm sieve. A soil sample was air-dried; particle size distribution was carried out according to the international pipette method of [75]. The pH value was determined by a pH meter according to [76]. CaCO_3_ was carried out according to [77]. Mineral content was determined using the methods of [78], and estimated using inductively coupled plasma spectrometry (Ultima 2 JY Plasma). Murphy and Riley [79] described the method which determined phosphorus. Available metals in the soil were extracted according to the method of [80] using a mixture solution of diethylenetriaminepentaacetic acid 97% (DTPA) and ammonium bicarbonate (pH 7.6). Soil texture is clay containing sand, silt and clay with percentages of 13.9%, 27.4% and 58.7%, respectively. Soil characteristics were pH: 8.24, total and available P: (0.69% and 0.21%), available micronutrients (Fe: 0.239 ppm, Zn: 0.1425 ppm, Mn: 0.201 ppm), cations (K^+^:0.37%, Mg^2+^: 6.34%, Ca^2+^: 8.47%), organic matter: 1.24% and CaCO_3_: 4.98%.

Pea (*Pisum sativum* L.) seeds, obtained from Agricultural Research Center, Giza, Egypt, were surface disinfected by drenching in 3% sodium hypochlorite (NaOCl) solution for 10 min, washed several times and soaked in distilled water for 2 h, and then sowed in a 25 cm diam. plastic pot (10 seeds/pot) containing 2.5 kg of sterilized soil in a greenhouse under controlled conditions (10 h light/14 h dark cycle) (day/night) at 20 °C and irrigated regularly with water, then seedlings were thinned to 4 seedlings/pot after germination.

### 4.5. Arbuscular Mycorrhizal (AM) Fungal Inoculation, ZnFe_2_O_4_ NPs Treatment and Sample Collection

AM fungal inoculum was *Funneliformis mosseae*, *Funneliformis constrictum*, *Gigaspora margarita* and *Rhizophagus irregularis* that had been previously isolated from the rhizosphere of different plant species [16] and identified by the manual for identification of AM fungi [81,82]. The mixture of AM fungal spores (in equal proportions) was propagated with Sudan grass (*Sorghum sudanenses* Pers.) roots as an appropriate trap plant, using a sterilized 1:1:1 sand: vermiculite: perlite mixture as a substrate. After 5 months of growth (87% colonization index), the substrate was allowed to dry, the roots were cut, and the inoculums (consisting of at least 950 spores/100 g, infected root pieces, hyphae and substrate) were maintained until use. AM fungal inoculation was applied by placing 20 g of inoculum for inoculated plants. Control (non-AM) plants received 20 g sterilized soil besides filtered washings of an equal amount of AM soil inoculum to provide the same associated microorganisms without AM spores.

After 12 days from sowing, ZnFe_2_O_4_ NPs were added to the soil at a concentration of 5 μM. Leaching of Zn and Fe ions from the parent ZnFe_2_O_4_ NPs into the soil was 4.245 and 7.7% after 48 h, respectively. The control treatments were applied with tap water. Hence, there are four treatments as follows: control plants (non-treated with AM fungi or ZnFe_2_O_4_ NPs), AM-inoculated (AM), ZnFe_2_O_4_ NP-treated (5 μM) and a combination of AM and 5 μM ZnFe_2_O_4_ NPs. Each treatment was replicated three times. Wariness should be taken in watering the plants to avoid the inoculum washing out of the soil during the first few days after inoculation and also to avoid leaching of NPs outside the pots. After 40 days from the ZnFe_2_O_4_ NP application, the plants were harvested and cleaned, then shoots and roots were saved for further experiments. Sub-samples of fresh fibrous roots were taken to evaluate root colonization.

### 4.6. Measurements

#### 4.6.1. Growth Traits

Fresh weights (FW) of pea shoots and roots were recorded, and their dry weights (DW) were obtained by drying them in an oven at 70 °C for 3 days. Additionally, the leaf number was recorded as the mean value of all plant leaves divided by the number of total plants for each treatment.

#### 4.6.2. AM Fungal Colonization Percentage

Fresh fine roots of AM-inoculated pea plants were cut into 1-cm segments and soaked in 20% KOH solution for 3 days at room temperature. The KOH was rinsed off and the root segments were acidified in 1% HCl overnight and subsequently stained with trypan blue for 24 h [83]. Pea roots were then destained in a 1% HCl/glycerol mixture. Root segments were placed on slides, and the colonization components were determined microscopically according to the method of [84].

#### 4.6.3. Photosynthetic Pigment Contents

A known pea leaf fresh weight (200 mg) was cut into small pieces and homogenized in 10 mL of 85% acetone. The homogenate was centrifuged at 4000× *g* for 15 min. The absorbance (A) of the collected supernatant (663, 644, and 452.5 nm) was measured [85] via a UV-visible spectrophotometer, RIGOL (Model Ultra-3660). The following equations were applied to calculate the pigment content of samples in terms of mg g^−1^ FW:

Chlorophyll a = (10.3 A663 − 0.918 A644) × *V/*(1000 × *W*),

Chlorophyll b = (19.7 A644 − 3.870 A663) × *V/*(1000 × *W*),

Carotenoids = (4.2 A452.5) − (0.0264 Chl. a + 0.426 Chl. b) × *V/*(1000 × *W*).

where *V* is the volume of 85% (*v*/*v*) acetone (mL), and *W* is the fresh weight (FW) of sample (g).

#### 4.6.4. Fe and Zn Contents and Translocation Factor (TF)

After mineralizing 0.1 g DW of the shoots and roots, the pea samples were placed in concentrated HNO_3_-HCl (1:3) for 12 h at ambient temperature, then for 2 h at 180 °C; Fe and Zn were then extracted [86]. Fe and Zn plant uptake was assessed using inductively coupled plasma mass spectrometry (ICP–MS) (Ultima 2 JY Plasma) at the Central Lab of Agricultural Research, Faculty of Agriculture, Zagazig University, Zagazig, Egypt. The Fe and Zn concentrations in the samples were obtained according to the following equation:

$$\mathrm{Concentration}\;\mathrm{of}\;\mathrm{sample}=\frac{\mathrm{ICP}-\mathrm{MS}\;\mathrm{reading}\;\times \;\mathrm{total}\;\mathrm{volume}\;\left(\mathrm{mL}\right)}{\mathrm{Weight}\;\mathrm{of}\;\mathrm{sample}\;\left(g\right)}$$

The transition of Fe and Zn from root to shoot was assessed in terms of translocation factor (TF) [87] and defined according to the following equation:

$$\mathrm{Translocation}\;\mathrm{Factor}\;\left(\mathrm{TF}\right)=\frac{\mathrm{Concentration}\;\mathrm{in}\;\mathrm{plant}\;\mathrm{shoot}}{\;\mathrm{Concentration}\;\mathrm{in}\;\mathrm{plant}\;\mathrm{root}}$$

### 4.7. Statistical Analysis

All experiments were accomplished with randomized sets including at least triplicate sampling. All data were subjected to the two-way analysis of variance using the SPSS version 15.0 statistical program for windows to determine the effects of AM, ZnFe_2_O_4_ NP and AM × ZnFe_2_O_4_ NP interaction on different measured parameters. The values were presented as means ± SE (standard error) by Duncan’s multiple range test as post hoc multiple comparisons. The significance of difference between mean values was expressed at a 95% confidence level when the treatments’ mean was compared with control [88]. In the graphical presentations, significant changes concerning the control levels were indicated by different letters.

## 5. Conclusions

The current study focuses on the impact of ZnFe_2_O_4_ NPs on AM fungal colonization and pea plant growth and the role of AM fungal hyphae in their uptake. Firstly, ZnFe_2_O_4_NPs were synthesized, and then their translocation into pea plant bodies was assessed by ICP–MS analysis. The outcomes of the current study show that ZnFe_2_O_4_ NPs positively affected pea plants as compared to the control, with more enhancements owing to AM fungal inoculation. Additionally, ZnFe_2_O_4_ NPs are taken up by the roots and migrate to the leaves, which led to stimulations in mineral uptake and some plant growth criteria (i.e., pigment content, shoots and roots’ fresh and dry weights) without phytotoxic effects. Overall, these findings suggest the safe use of ZnFe_2_O_4_ NPs coupled with AM fungi in nanoagricultural applications.

## Figures and Tables

**Table 1:** Significance levels (F-values) of treatments and treatment interactions of some measured variables based on a two-way ANOVA analysis.

Variables	AM	ZnFe_2_O_4_ NPs	AM+ ZnFe_2_O_4_ NPs
Shoot FW	40.47 *	14.39 *	0.167 ns
Root FW	81.49 *	10.56 *	6.39 *
Shoot DW	39.85 *	36.46 *	11.81 ns
Root DW	65.56 *	43.86 *	1.06 ns
R/S ratio	26.02 *	2.89 ns	32.12 *
Chlorophyll a	4.269 ns	2.038 ns	0.559 ns
Chlorophyll b	50.05 *	8.788 *	15.658 *
Carotenoids	22.16 *	4.93 ns	9.48 *
Total Chlorophyll	13.3 *	5.886 *	0.079 ns
Total pigments	7.63 *	5.82 *	2.16 ns
Shoot Zn conc	7.956 *	138.8 *	0.213 ns
Root Zn conc	6.74 *	54.56 *	0.207 ns
Total Zn conc	7.49 *	101.15 *	0.211 ns

Significance levels: *, significant; ns, non-significant effect.

**Table 2:** ZnFe_2_O_4_ NPs and AM fungal effects on pigment content of pea plant leaves.

Treatment	Chlorophyll a	Chlorophyll b	Carotenoids	Total Chlorophyll	Total Pigments
	(mg g^−1^ FW)	(mg g^−1^ FW)	(mg g^−1^ FW)	(mg g^−1^ FW)	(mg g^−1^ FW)
Control	1.441 ± 0.076 b	0.812 ± 0.043 bc	0.297 ± 0.016 b	2.252 ± 0.12 b	2.549 ± 0.135 b
AM	1.497 ± 0.079 ab	0.976 ± 0.052 b	0.270 ± 0.014 b	2.473 ± 0.13 b	2.743 ± 0.145 b
ZnFe_2_O_4_ NPs	1.549 ± 0.082 ab	0.759 ± 0.04 c	0.382 ± 0.02 a	2.309 ± 0.12 b	2.691 ± 0.142 b
AM+ ZnFe_2_O_4_ NPs	1.729 ± 0.091 a	1.342 ± 0.071 a	0.256 ± 0.014 b	3.071 ± 0.16 a	3.327 ± 0.176 a

Control: represents non-treated pea plants; AM: represents pea plants inoculated with AM fungi; Zn/Fe NPs: pea plants treated with ZnFe_2_O_4_ NPs and AM + Zn/Fe NPs: represents pea plants dually treated with AM fungi and ZnFe_2_O_4_ NPs. Data are the mean of three replicates ± standard error (n = 3). Values in each column followed by the same letter(s) are not significantly different at *p* ≤ 0.05 (Duncan’s multiple range test). FW represents fresh weight.

## Data Availability

No new data were created or analyzed in this study. Data sharing is not applicable to this article.

## References

[1] Spielman D.J., Malik S.J., Dorosh P., Ahmad N., Spielman D.J., Malik S.J., Dorosh P., Ahmad N. (2017). Chapter 1. Agriculture and the Rural Economy in Pakistan. Agriculture and the Rural Economy in Pakistan: Issues, Outlooks, and Policy Priorities.

[2] FAO (2014). FAOSTAT.

[3] Selim Y.A., Azb M.A., Ragab I., Abd El-Azim M.H.M. (2020). Green Synthesis of Zinc Oxide Nanoparticles Using Aqueous Extract of *Deverra tortuosa* and their Cytotoxic Activities. Sci. Rep..

[4] Dimkpa C.O., Bindraban P.S. (2018). Nanofertilizers: New products for the Industry?. J. Agric. Food Chem..

[5] Abdel Latef A.A., Zaid A., Abu Alhmad M.F., Abdelfattah K.E. (2020). The Impact of priming with Al_2_O_3_ nanoparticles on growth, pigments, osmolytes, and antioxidant enzymes of Egyptian roselle (*Hibiscus sabdariffa* L.) cultivar. Agronomy.

[6] Baranowska-Wójcik E., Szwajgier D., Oleszczuk P., Winiarska-Mieczan A. (2019). Effects of titanium dioxide nanoparticles exposure on human health—A Review. Biol. Trace Elem. Res..

[7] Abdel Latef A.A., Alhmad M.F., Abdelfattah K.E. (2017). The possible roles of priming with ZnO nanoparticles in mitigation of salinity stress in Lupine (*Lupinus termis*) plants. J. Plant Growth Regul..

[8] Abdel Latef A.A., Srivastava A.K., Abdel-sadek M.S., Kordrostam M., Tran L.-S.P. (2018). Titanium dioxide nanoparticles improve growth and enhance tolerance of broad bean plants under saline conditions. Land Degrad. Dev..

[9] Rajput V., Minkina T., Mazarji M., Shende S., Sushkova S., Mandzhieva S., Burachevskaya M., Chaplygin V., Singh A., Jatav H. (2020). Accumulation of nanoparticles in the soil-plant systems and their effects on human health. Ann. Agric. Sci..

[10] Wang F., Liu X., Shi Z., Tong R., Adams C., Shi X. (2016). Arbuscular mycorrhizae alleviate negative effects of zinc oxide nanoparticle and zinc accumulation in maize plants -A soil microcosm experiment. Chemosphere.

[11] Berg G. (2009). Plant-microbe interactions promoting plant growth and health: Perspectives for controlled use of microorganisms in agriculture. Appl. Microbiol. Biotechnol..

[12] Cao J., Feng Y., Lin X., Wang J., Xie X. (2017). Iron oxide magnetic nanoparticles deteriorate the mutual interaction between arbuscular mycorrhizal fungi and plant. J. Soils Sediments.

[13] Begum N., Qin C., Ahanger M.A., Raza S., Khan M.I., Ashraf M., Ahmed N., Zhang L. (2019). Role of arbuscular mycorrhizal fungi in plant growth regulation: Implications in abiotic stress tolerance. Front Plant Sci..

[14] Abdelhameed R.E., Metwally R.A. (2019). Alleviation of cadmium stress by arbuscular mycorrhizal symbiosis. Int. J. Phytoremediation.

[15] Metwally R.A. (2020). Arbuscular mycorrhizal fungi and *Trichoderma viride* cooperative effect on biochemical, mineral content, and protein pattern of onion plants. J. Basic Microbiol..

[16] Metwally R.A., Soliman S.A., Abdel Latef A.A., Abdelhameed R.E. (2021). The Individual and interactive role of arbuscular mycorrhizal fungi and *Trichoderma viride* on growth, protein content, amino acids fractionation, and phosphatases enzyme activities of onion plants amended with fish waste. Ecotoxicol. Environ. Saf..

[17] Tian H., Kah M., Kariman K. (2019). Are Nanoparticles a Threat to Mycorrhizal and Rhizobial Symbioses? A Critical Review. Front. Microbiol..

[18] Watts-Williams S.J., Patti A.F., Cavagnaro T.R. (2013). Arbuscular mycorrhizas are beneficial under both deficient and toxic soil zinc conditions. Plant Soil..

[19] Manceau A., Nagy K.L., Marcus M.A., Lanson M., Geoffroy N., Jacquet T., Kirpichtchikova T. (2008). Formation of metallic copper nanoparticles at the soil-root interface. Environ. Sci. Technol..

[20] Cao J., Feng Y., Lin X., Wang J. (2016). Arbuscular mycorrhizal fungi alleviate the negative effects of iron oxide nanoparticles on bacterial community in rhizospheric soils. Front. Environ. Sci..

[21] Feng Y., Cui X., He S., Dong G., Chen M., Wang J., Lin X. (2013). The role of metal nanoparticles in influencing arbuscular mycorrhizal fungi effects on plant growth. Environ. Sci. Technol..

[22] Dubchak S., Ogar A., Mietelski J.W., Turnau K. (2010). Influence of silver and titanium nanoparticles on arbuscular mycorrhiza colonization and accumulation of radiocaesium in *Helianthus annuus*. Span. J. Agric. Res..

[23] Abu-Elsaad N.I., Abdelhameed R.E. (2019). Copper ferrite nanoparticles as nutritive supplement for cucumber plants grown under hydroponic system. J. Plant Nutri..

[24] Groenou A.B.V., Bongers P.F., Stuyts A.L. (1968). Magnetism, microstructure and crystal chemistry of spinel ferrites. Mater. Sci. Eng..

[25] Nakamura T., Demidzu H., Yamada Y. (2008). Synthesis and magnetic study on Mg^2+^-substituted Li–Mn spinel oxides. J. Phys. Chem. Solids..

[26] Elayakumar K.A., Manikandan A., Dinesh K., Thanrasu K., Kanmani Raja R., Thilak Kumar Y., Slimani S.K., Jaganathan A.B. (2019). Enhanced magnetic property and antibacterial biomedical activity of Ce^3+^doped CuFe_2_O_4_ spinel nanoparticles synthesized by sol-gel method. J. Magn. Magn. Mater..

[27] Zablotskii V., Polyakova T., Lunov O., Dejneka A. (2016). How a high-gradient magnetic field could affect cell life. Sci. Rep..

[28] Tombuloglu H., Slimani Y., Tombuloglu G., Almessiere M., Baykal A., Ercan I., Sozeri H. (2019). Tracking of NiFe_2_O_4_ nanoparticles in barley (*Hordeum vulgare* L.) and their impact on plant growth, biomass, pigmentation, catalase activity, and mineral uptake. Environ. Nanotechnol. Monit. Mon..

[29] Nisticò R., Cesano F., Garello F. (2020). Magnetic materials and systems: Domain structure visualization and other characterization techniques for the application in the materials science and biomedicine. Inorganics.

[30] Boon M.S., Serena Saw W.P., Jaafar M. (2012). Magnetic, dielectric and thermal stability of Ni–Zn ferrite-epoxy composite thin films for electronic applications. J. Magn. Magn. Mater..

[31] Okoroh D.O., Aisida S.O., Asogwa P.U. (2018). Synthesis and Characterization of Biopolymer capped Zinc ferrite nanoparticles by a thermal treatment method. IOSR-JAP.

[32] López-Moreno M.L., Avilés L.L., Pérez N.G., Irizarry B.Á., Perales O., Cedeno-Mattei Y., omán F. (2016). Effect of cobalt ferrite (CoFe_2_O_4_) nanoparticles on the growth and development of *Lycopersicon lycopersicum* (tomato plants). Sci. Total Environ..

[33] Nair R., Varghese S.H., Nair B.G., Maekawa T., Yoshida Y., Kumar D.S. (2010). Nanoparticulate material delivery to plants. Plant Sci..

[34] Maphosa Y., Jideani V. (2017). The Role of legumes in human nutrition In: Functional food-improve health through adequate food. Functional Food-Improve Health through Adequate Food.

[35] Cullity B.D. (1959). Elements of X-ray Diffraction.

[36] Cullity B.D. (1956). Elements of X-ray Diffraction.

[37] Somvanshi S.B., Khedkar M.V., Kharat P.B., Jadhav K.M. (2020). Influential diamagnetic magnesium (Mg^2+^) ion substitution in nano-spinel zinc ferrite (ZnFe_2_O_4_): Thermal, structural, spectral, optical and physisorption analysis. Ceram Int..

[38] Mazen S.A., Abu-Elsaad N.I. (2012). Structural and some magnetic properties of manganese-substituted lithium ferrites. J. Magn. Magn. Mater..

[39] Marschner H. (2011). Marschner’s Mineral Nutrition of Higher Plants.

[40] Rengel Z. (2015). Availability of Mn, Zn and Fe in the rhizosphere. J. Soil Sci. Plant Nutr..

[41] Hefferon K. (2019). Biotechnological approaches for generating zinc-enriched crops to combat malnutrition. Nutrients.

[42] Myers S.S., Wessells K.R., Kloog I., Zanobetti A., Schwartz J. (2015). Effect of increased concentrations of atmospheric carbon dioxide on the global threat of zinc deficiency: A modelling study. Lancet Glob. Health..

[43] Mayer J.E., Pfeiffer W.H., Beyer P. (2008). Biofortified crops to alleviate micronutrient malnutrition. Curr. Opin. Plant Biol..

[44] Subbaiah L.V., Prasad T.N., Krishna T.G., Sudhakar P., Reddy B.R., Pradeep T. (2016). Novel effects of nanoparticulate delivery of zinc on growth, productivity, and zinc biofortification in maize (*Zea mays* L.). J. Agric. Food Chem..

[45] Šoltysová B., Danilovič M. (2011). Tillage in relation to distribution of nutrients and organic carbon in the soil. Agriculture (Poľnohospodárstvo).

[46] Jacoby R., Peukert M., Succurro A., Koprivova A., Kopriva S. (2017). The Role of Soil Microorganisms in Plant Mineral Nutrition—Current Knowledge and Future Directions. Front. Plant Sci..

[47] Hong F., Yang P., Gao F., Liu C., Zheng L., Yang F. (2005). Effect of nano-anatase TiO_2_ on spectral characterization of photosystem particles from spinach. Chem. Res. China Univ..

[48] Sebastian R.M., Vijayalakshmy K.C., Lakshmi S., Saramma A.V., Mohammed E.M. (2014). Effect of Zinc Ferrite nanoparticles on the growth of *Chlorella pyrenoidosa*. Res. J. Pharm. Biol. Chem. Sci..

[49] Singh J., Kumar S., Alok A., Upadhyay S.K., Rawat M., Tsang D.C.W., Bolan N., Kim K.-H. (2019). The potential of green synthesized zinc oxide nanoparticles as nutrient source for plant growth. J. Clean. Prod..

[50] Rivero-Montejo S.D.J., Vargas-Hernandez M., TorresPacheco I. (2021). Nanoparticles as Novel Elicitors to Improve Bioactive Compounds in Plants. Agriculture.

[51] Tombuloglu H., Slimani Y., Tombuloglu G., Alshammari T., Almessiere M., Korkmaz A.Y., Baykal A.S., Samia A.C. (2020). Engineered magnetic nanoparticles enhance chlorophyll content and growth of barley through the induction of photosystem genes. Environ. Sci. Pollut. Res..

[52] Liu X., Wang F., Shi Z., Tong R., Shi X. (2015). Bioavailability of Zn in ZnO nanoparticles-spiked soil and the implications to maize plants. J. Nanopart. Res..

[53] Wang F., Adams C.A., Shi Z., Sun Y. (2018). Combined effects of ZnO NPs and Cd on sweet sorghum as influenced by an arbuscular mycorrhizal fungus. Chemosphere.

[54] Wang X., Yang X., Chen S., Li Q., Wang W., Hou C., Gao X., Wang L., Wang S. (2016). Zinc oxide nanoparticles affect biomass accumulation and photosynthesis in Arabidopsis. Front. Plant Sci..

[55] Metwally R.A., Al-Amri S.M. (2019). Individual and interactive role of *Trichoderma viride* and arbuscular mycorrhizal fungi on growth and pigment content of onion plants. Lett. Appl. Microbiol..

[56] Abdel-Fattah G.M., Asrar A.W.A. (2012). Arbuscular mycorrhizal fungal application to improve growth and tolerance of wheat (*Triticum aestivum* L.) plants grown in saline soil. Acta Physiol. Plant..

[57] Abdel Latef A.A., Chaoxing H. (2011). Effect of arbuscular mycorrhizal fungi on growth, mineral nutrition, antioxidant enzymes activity and fruit yield of tomato grown under salinity stress. Sci. Hortic..

[58] Metwally R.A., Abdelhameed R.E. (2019). Impact of Ridomil, Bavistin and Agrothoate on arbuscular mycorrhizal fungal colonization, biochemical changes and potassium content of cucumber plants. Ecotoxicology.

[59] Abdel Latef A.A., Chaoxing H. (2014). Does the inoculation with *Glomus mosseae* improve salt tolerance in pepper plants?. J. Plant Growth Regul..

[60] Abdelhameed R.E., Metwally R.A. (2018). Mitigation of salt stress by dual application of arbuscular mycorrhizal fungi and salicylic acid. Agrochimica.

[61] Metwally R.A., Abdelhameed R.E. (2018). Synergistic effect of arbuscular mycorrhizal fungi in growth and physiology of salt-stressed *Trigonella foenum-graecum* plants. Biocatal. Agric. Biotechnol..

[62] Wang Y., Wang M., Li Y., Wu A., Huang J. (2018). Effects of arbuscular mycorrhizal fungi on growth and nitrogen uptake of *Chrysanthemum morifolium* under salt stress. PLoS ONE.

[63] Koomen I., Firestone M.K., Giller I. (1990). Mycorrhizal infection of clover is delayed in soils contaminated with heavy metals from past sewage sludge applications. Soil. Biol. Biochem..

[64] Liu A., Hamel C., Hamilton R.I., Ma B.L., Smith D.L. (2000). Acquisition of Cu, Zn, Mn and Fe by mycorrhizal maize (*Zea mays* L.) grown in soil at different P and micronutrient levels. Mycorrhiza.

[65] Ibiang Y.B., Innami H., Sakamoto K. (2018). Effect of excess zinc and arbuscular mycorrhizal fungus on bioproduction and trace element nutrition of Tomato (*Solanum lycopersicum* L. cv. Micro-Tom). J. Soil Sci. Plant Nutr..

[66] Sharma N., Yadav K., Aggarwal A. (2016). Growth response of two Phaseolus mungo L. cultivars induced by arbuscular mycorrhizal fungi and Trichoderma viride. Int. J. Agron..

[67] Satti S.H., Raja N.I., Javed B., Akram A., Mashwani Z.U.R., Ahmad M.S. (2021). Titanium dioxide nanoparticles elicited agromorphological and physicochemical modifications in wheat plants to control *Bipolaris sorokiniana*. PLoS ONE.

[68] Jalali M., Ghanati F., Modarres-Sanavi A.M., Khoshgoftarmanesh A.H. (2017). Physiological effects of repeated foliar application of magnetite nanoparticles on maize plants. J. Agron. Crop Sci..

[69] Evelin H., Devi T.S., Gupta S., Kapoor R. (2019). Mitigation of Salinity Stress in Plants by Arbuscular Mycorrhizal Symbiosis: Current Understanding and New Challenges. Front. Plant Sci..

[70] Kong H., Song J., Jang J. (2010). One-step fabrication of magnetic γ-Fe_2_O_3_/polyrhodanine nanoparticles using in situ chemical oxidation polymerization and their antibacterial properties. Chem. Commun..

[71] Zhao L., Hernandez-Viezcas J., Peng B., Munoz B., Keller A., Peralta-Videa J.R., Gardea-Torresdey J. (2013). Zno nanoparticle fate in soil and zinc bioaccumulation in corn plants (*Zea mays*) influenced by alginate. Environ. Sci. Process. Impacts..

[72] Zhang P., Ma Y., Zhang Z., He X., Zhang J., Guo Z., Tai R., Zhao Y., Chai Z. (2012). Biotransformation of ceria nanoparticles in cucumber plants. ACS Nano.

[73] Lv J., Zhang S., Luo L., Zhang J., Yangc K., Christie P. (2015). Accumulation, speciation and uptake pathway of ZnO nanoparticles in maize. Environ. Sci. Nano.

[74] Tommaso G., Alberto F., Lucia G., Lucia N., Giulia G., Fausto R., Rea C., Alberto P. (2012). Response of tomato plants exposed to treatment with nanoparticles. Environ. Qual..

[75] Piper C.S. (1950). Soil and Plant Analysis.

[76] Chapman H.D., Pratt P. (1982). Methods of Analysis for Soils, Plants and Water.

[77] Jackson M.L. (1967). Soil Chemical Analysis.

[78] Nation J.L., Robinson F.A. (1971). Concentration of some major and trace elements in honeybee, royal jelly and pollen determined by atomic absorption spectrophotometer. J. Apicult. Res..

[79] Murphy J., Riley J. (1962). A modified single solution method for the determination of phosphate in natural waters. Anal. Chim Acta.

[80] Soltanpour P.N. (1991). Determination of nutrient element availability and elemental toxicity by the AB-DTPA soil test and ICPS. Adv. Soil Sci..

[81] Schenck N.C., Smith G.S. (1982). Additional new and unreported species of mycorrhizal fungi (Endogonaceae) from Florida. Mycologia.

[82] Walker C., Schüßler A. (2010). The Glomeromycota: A Species List with New Families and New Genera.

[83] Phillips J., Hayman D. (1970). Improved procedures for clearing roots and staining parasitic and vesicular arbuscular mycorrhizal fungi for rapid assessment of infection. Trans. Br. Mycol. Soc..

[84] Trouvelot A., Kough J.L., Gianinazzi-Pearson V., Gianinazzi-Pearson V., Et Gianinazzi S. (1986). Measure des taux de mycorhization VA d, UN system radiculaire. Recherche de methodes d’estimation ayant une signification fonctionnelle. Physiological and Genetical Aspects of Mycorrhizae.

[85] Metzner H., Rau H., Senger H. (1965). Untersuchungen Zur Synchronisierbarkeit einzelner Pigment-Mangel Mutanten Von Chlorella. Planta.

[86] Karaca A. (2004). Effect of organic wastes on the extractability of cadmium, copper, nickel, and zinc in soil. Geoderma.

[87] Li M.S., Luo Y.P., Su Z.Y. (2007). Heavy metal concentrations in soils and plant accumulation in a restored manganese mineland in Guangxi, South China. Environ. Pollut..

[88] Levesque R. (2007). SPSS Programming and Data Management: A Guide for SPSS and SAS Users.

